# Whole-genome sequencing of *Mycobacterium tuberculosis* from Cambodia

**DOI:** 10.1038/s41598-022-10964-9

**Published:** 2022-05-11

**Authors:** Konstantin Edokimov, Yoshiyuki Yamada, Chhavarath Dary, Qing Hao Miow, Li-Yang Hsu, Rick Twee-Hee Ong, Vonthanak Saphonn

**Affiliations:** 1 Saw Swee Hock School of Public Health, National University of Singapore and National University Health System, 12 Science Drive 2, #10-01, Singapore, 117549 Singapore; 2 University of Health Sciences, #73, Preah Monivong Blvd, Sangkat Sras Chak, Khan Daun Penh, Phnom Penh, Cambodia; 3 Department of Medicine, Yong Loo Lin School of Medicine, National University of Singapore, Singapore, Singapore

**Keywords:** Tuberculosis, Epidemiology

## Abstract

Cambodia has one of the highest tuberculosis (TB) incidence rates in the WHO Western Pacific region. Remarkably though, the prevalence of multidrug-resistant TB (MDR-TB) remains low. We explored the genetic diversity of *Mycobacterium tuberculosis (MTB)* circulating in this unique setting using whole-genome sequencing (WGS). From October 2017 until January 2018, we collected one hundred sputum specimens from consenting adults older than 21 years of age, newly diagnosed with bacteriologically confirmed TB in 3 districts of Phnom Penh and Takeo provinces of Cambodia before they commence on their TB treatment, where eighty *MTB* isolates were successfully cultured and sequenced. Majority of the isolates belonged to Lineage 1 (Indo-Oceanic) (69/80, 86.25%), followed by Lineage 2 (East Asian) (10/80, 12.5%) and Lineage 4 (Euro-American) (1/80, 1.25%). Phenotypic resistance to both streptomycin and isoniazid was found in 3 isolates (3/80, 3.75%), while mono-resistance to streptomycin and isoniazid was identical at 2.5% (N = 2 each). None of the isolates tested was resistant to either rifampicin or ethambutol. The specificities of genotypic prediction for resistance to all drugs tested were 100%, while the sensitivities of genotypic resistance predictions to isoniazid and streptomycin were lower at 40% (2/5) and 80% (4/5) respectively. We identified 8 clusters each comprising of two to five individuals all residing in the Takeo province, making up half (28/56, 50%) of all individuals sampled in the province, indicating the presence of multiple ongoing transmission events. All clustered isolates were of Lineage 1 and none are resistant to any of the drugs tested. This study while demonstrating the relevance and utility of WGS in predicting drug resistance and inference of disease transmission, highlights the need to increase the representation of genotype–phenotype TB data from low and middle income countries in Asia and Africa to improve the accuracies for prediction of drug resistance.

## Introduction

Cambodia, situated at the crossroads between countries in WHO South-East Asia and Western Pacific regions, had one of the highest TB incidence and mortality rates of 326 and 21.6 per 100,000 respectively. The percentage of multidrug-resistant TB (MDR-TB) in Cambodia had however remained relatively low at 1.8% among new and 11% among previously treated TB cases as was found in the National Tuberculosis Drug Resistance Survey in 2000–2001^1,2^.

Increasingly, molecular techniques, such as whole-genome sequencing (WGS) had been used extensively in multiple studies to provide rapid diagnosis, drug susceptibility profiling and in understanding TB transmissions^3,4^. However, accurate drug susceptibility profiling require the exact knowledge of the genetic determinants of drug resistance in *Mycobacterium tuberculosis* (MTB)^5,6^. The lack of low and middle income countries within Asia and Africa in global TB initiatives including the identification of genetic mutations associated with drug resistance is therefore especially concerning^7,8^. Extrapolation of findings from either low TB burden countries or high drug-resistant TB settings is problematic due to a bias towards certain resistance mutations and dominance of specific lineages that might differ across settings. In particular, little is known about the genetic diversity and drug resistance determinants of MTB found in Cambodia^9^. The role of WGS in detecting TB transmission and for investigation of outbreaks had been demonstrated in multiple studies^4,10^ but had never been explored in Cambodia.

In this study, we thus explore how insights obtained with whole-genome sequencing of MTB circulating in the unique setting of a high TB burden with low MDR-TB prevalence in Cambodia might inform TB control efforts, through examining the genetic diversity, variants associated with resistance to TB drugs and inference of active transmission in the community.

## Results

### MTB strains characteristics

**Figure 1:**
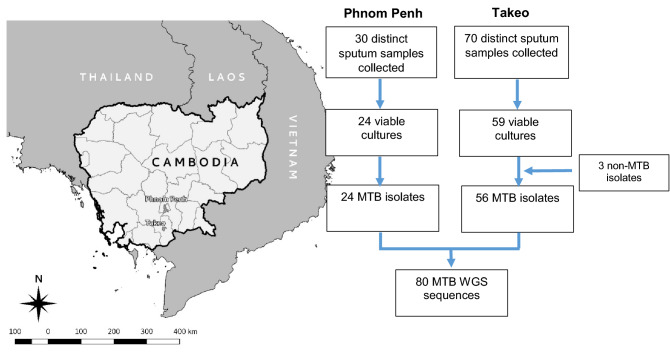
Map and flowchart illustrating the sampling collections of Phnom Penh and Takeo within Cambodia and number of samples collected. The map was created with QGIS software version 3.8.0 distributed under the Creative Commons Attribution-ShareAlike 3.0 licence (CC BY-SA) (https://www.qgis.org/en/site/forusers/download.html) using shape files generated from OpenStreetMap data which are licensed under the Open Database 1.0 License (www.openstreetmap.org).

One hundred sputum samples were collected from new, bacteriologically confirmed, pulmonary TB cases between October 2017 and January 2018 in three operational districts of Cambodia: Daun-Keo in the Takeo province, Dangkao and Pou Senchey within Phnom Penh capital (Fig. 1). Eighty-three mycobacteria isolates were successfully cultured and sequenced. The sequence reads from 80 samples were successfully mapped to the H37Rv reference genome with an average read depth of 251X and mean genome coverage of 98.9%. Based on Kraken analysis, the three poorly-mapped samples were identified to be from non-tuberculous mycobacteria (NTM) species of *M. sinense* and *M. terrae* and of *Mycobacterium abscessus* species and were therefore excluded from further analysis (Fig. 1).

### Phenotypic and genotypic antimicrobial resistance

Majority of the isolates (73/80, 91.25%) were phenotypically sensitive to all TB drugs tested, while a handful (7/80, 8.75%) were resistant to at least one TB drug (isoniazid or streptomycin) including 3 isolates from Phnom Penh that were resistant to both drugs (Table 1). High accuracies were obtained for the genotypic-phenotypic correlation of resistance predictions (rifampicin = 80/80, 100%; isoniazid = 77/80, 96.25%; ethambutol = 80/80, 100%; streptomycin = 78/80, 97.5%), where known genotypic mutations associated with isoniazid resistance were found in *katG* (S315T) and the *fabG1-inhA* promoter region (C-15 T); while mutations associated with streptomycin resistance were found in the *rpsL* (K88R and K43R) and *rrs* (517c > t) genes (Table 1). In all of the phenotypic drug-susceptible isolates, we did not identify the presence of any known genotypic mutations associated with resistance in the genes assessed. Thus, the specificities of the genotypic prediction of resistance for each of the TB drugs tested were 100%. However, the sensitivities of genotypic resistance predictions to isoniazid and streptomycin were lower at 40% and 80% respectively.

**Table 1:** Correlating genotypic variants with phenotypic resistance to TB drugs, grouped by lineages.

Isolate/lineage/location	Phenotype/genotype	
	Isoniazid	Streptomycin
cam023/L1.1.1/Takeo	S	R/No known resistance mutation identified Mutations found: gid_K163E whiB6_Q94E
cam092/L1.1.1/Phnom Penh	R/No known resistance mutation identified Mutations found: katG_R463L ndh_V18G	R/rrs r.517c > t
cam036/L1.2.1/Takeo	R/No known resistance mutation identified Mutations found: katG_R463L ahpC_H93R	S
cam062/L2.2.1/Phnom Penh	S	R/rpsL_K88R
cam094/L2.2.1/Takeo	R/inhA_C-15T	S
cam027/L2.2.1/Phnom Penh	R/katG_S315T	R/rpsL_K88R
cam063/L2.2.1/Phnom Penh	R/No known resistance mutation identified Mutations found: katG_R463L katG_R128Q	R/rpsL_K43R

For the additional non-synonymous mutations identified within genes associated with resistance, katG_R463L had been reported not to be associated with isoniazid resistance: *R* resistant, *S* susceptible.

For phenotypic-resistant isolates with no known resistance genetic mutations identified, we assessed the non-synonymous mutations identified with the “Catalogue of mutations in *Mycobacterium tuberculosis* complex and their associated with drug resistance” from WHO^11^. For streptomycin, the two mutations: *gid_K163E* and *whiB6_Q94E* were classified within the category of Group 3: Uncertain significance in the WHO catalogue, where the variant *gid_K163E* was observed once in susceptible strains, and none in resistant strains, while *whiB6_Q94E* was seen in 73 susceptible strains and twice in resistant strains. For isoniazid, the variant *katG_R463L* was observed to be present in all 3 discordant isolates, but this variant was classified within the category of Group 5: Not associated with resistance in the WHO catalogue. Another variant *katG_R128Q* identified was listed under Group 3: Uncertain significance, where the variant was observed once in resistant strains and none in susceptible strains. The two remaining variants: *ndh_V18G* and *ahpC_H93R* were not assessed in the WHO catalogue though the variant *ndh_V18A* was found in Group 5: Not associated with resistance within the WHO catalogue.

### MTB population structure

**Figure 2:**
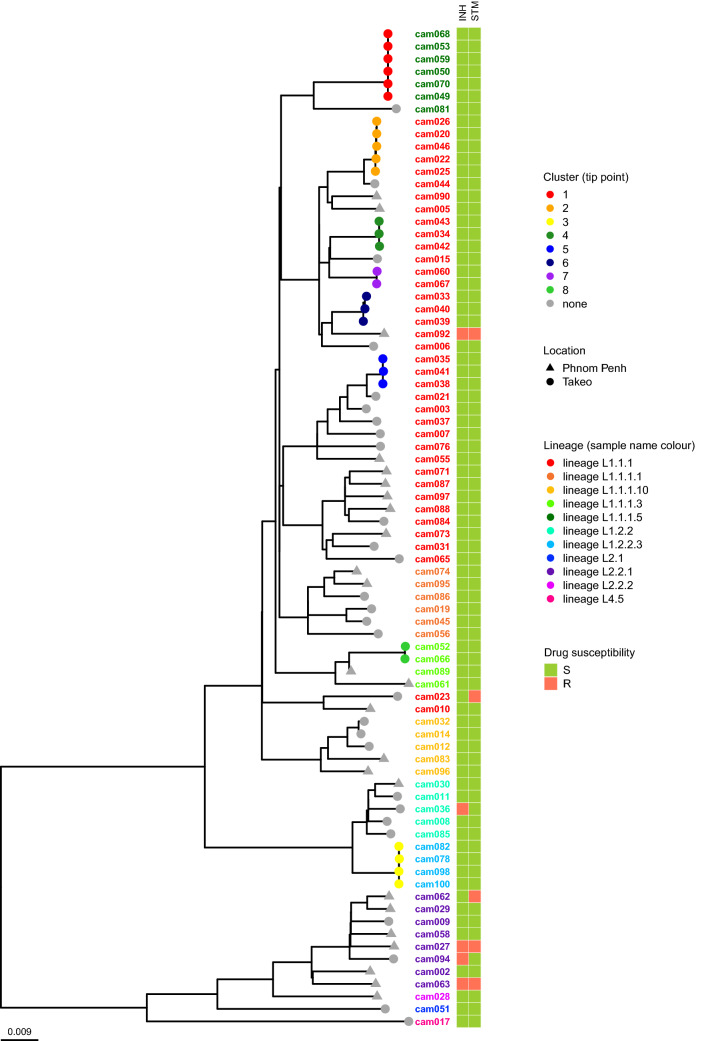
Maximum likelihood phylogenetic tree for the 80 MTB isolates in this study. The tree was constructed based on 12,353 SNPs extracted from the 80 MTB isolates, excluding SNPs in the PE/PPE and repeat regions. The tree is annotated (from left to right) to illustrate the location where isolate was collected (circle and triangle representing Phnom Penh and Takeo respectively), and if isolate was inferred to be clustered (colours representing the different clusters while grey indicates un-clustered isolates), sample identifier which is colour coded to represent SNP lineages (Lineage 1 are based on Netikul et al. scheme while Lineage 2 are based on 62-SNP Coll et al.), and two coloured columns indicating phenotypic susceptibility to isoniazid (INH) and streptomycin (STM) (green indicates susceptible, while red indicates resistant).

The inferred lineages of the MTB isolates were largely concordant across the 4 genotyping methods of (1) Coll et al. 62-SNP barcoding^12^; (2) Netikul et al. sub-lineage 1 prediction^13^; (3) spoligotypes from SpoTyping and (4) regions of deletions based on RD-Analyzer (Supplementary Table 1). Majority of the isolates belonged to Lineage 1 (Indo-Oceanic) (69/80, 86.25%), followed by Lineage 2 (East Asian) (10/80, 12.5%) and Lineage 4 (Euro-American) (1/80, 1.25%). The phylogeny inferred from the whole-genome SNPs supported the classification of the lineages and clades with the genotyping schemes (Fig. 2).

Most of the Lineage 2 isolates belonged to the L2.2.1 (Modern Beijing) sub-lineage (9/10, 90%) and were from Phnom Penh (7/10, 70%), while there was only one isolate that belonged to the L2.1 sub-lineage (proto-Beijing). The lineage-family inference of this lineage L2.1 isolate by its spoligotype was however unknown (SIT 246) based on a search in the SITVIT2 database^14^.

In this study, we identified isolates in only two of the three major Lineage 1 clades^12,13,15^, namely L1.1 and L1.2, with dominance of L1.1 (60/69, 87.0%). L1.3 was widespread in Africa, India and Thailand (specific sub-lineage of L1.3.2), while not found in Vietnam^13^.

Within L1.1, there are three major sublineages: L1.1.1-L1.1.3. Similar to the Vietnam isolates analysed in Netikul et al., we did not identify any isolates in this study belonging to L1.1.2 and L1.1.3. Within the L1.1.1 isolates, 22 isolates can be further clustered into L1.1.1.1 (6/22; 27.3%), L1.1.1.3 (4/22; 18.2%), L1.1.1.5 (7/22; 31.8%) and L1.1.1.10 (5/22; 22.7%). The distribution of the sub-lineages for L1.1 was thus found to correlate with distributions identified for Thailand and Vietnam as reported in Netikul et al.^13^, including a new clade of L1.1.1.10 found only in samples from Thailand and Vietnam.

Using the clade nomenclature as reported in Netikul et al., L1.2 can be delineated into 2 clades, where L1.2.1 mainly comprises of isolates collected from European countries, and L1.2.2 that is widespread across East Asia and Southeast Asia. In this study, we did not identify any isolates from L1.2.1, and found nine L1.2.2 isolates (9/69, 13.0%) where four can be further delineated into the sub-lineage of L1.2.2.3. The 1.2.2 sub-lineage isolates were predominantly from the Takeo province (8/9, 88.9%) (Fig. 2).

### Putative inferred active disease transmission through clustered isolates

Eight clusters were identified, each comprising of two to five individuals (Fig. 2), where the SNP differences between isolates ranged from zero to eight (Supplementary Table S2). All of the 28 clustered individuals resided within Takeo province, and made up half (28/56, 50%) of all individuals sampled in the province. None of the clustered isolates were found to be resistant to any of the TB drugs tested. In addition, all clustered isolates belonged to Lineage 1, where majority are from the sub-lineage L1.1.1 (24/28, 85.7%). Two clusters can be further delineated into sub-lineage L1.1.1.5 (cluster 1) and sub-lineage L1.1.1.3 (cluster 8). There are seven isolates in this study which have been identified to be of sub-lineage L1.1.1.5, all sampled within Takeo, and six of these are found in cluster 1. Similarly, there are three isolates identified to be from sub-lineage L1.1.1.3 where both isolates sampled from Takeo are found in cluster 8, while the remaining third isolate sampled from Phnom Penh differ by 58 SNPs with the clustered isolates. All four isolates belonging to the sub-lineage L1.2.2.3 in this study were found to group within cluster 3.

## Discussion

Our study presents the first reported results on the use of whole genome sequencing (WGS) of *Mycobacterium tuberculosis* (MTB) isolates in Cambodia. We found that, similar to a previous study performed on Cambodian MTB samples with lower-resolution molecular genotyping techniques of MIRU-VNTR and spoligotyping, collected almost a decade earlier^9^, the genetic diversity of MTB in Cambodia is still mainly driven by Lineage 1 Indo-Oceanic (EAI) and Lineage 2 East-Asia (Beijing). The high proportion of Lineage 1 MTB in Cambodia is perhaps not surprising since this is a common strain found circulating within countries in Southeast Asia, India and Bangladesh^16^. However, the resolution provided by WGS allows finer classification of the MTB isolates into sub-lineages which have been found to correlate with that found in Thailand and Vietnam, two countries that border Cambodia. This include five isolates belonging to lineage L1.1.1.10 that are found only in Thailand (N = 2) and Vietnam (N = 8) based on a phylogenetic study of 1,761 publicly available L1 MTB isolates^13^.

While the number of SNP differences between isolates do not directly indicate disease transmission, it is often employed in practice for inferring putative transmission links between TB cases^17^. Different SNP thresholds had been used in low- and high TB-burden settings to reflect the differences in transmission dynamics^18–21^. In our study, although 10 SNPs was used as the cut-off threshold, we found that there was only 1 pair of MTB isolates that differed at 8 SNPs, while the remaining pairs differed at less than 3 SNPs apart. One limitation of our study was, given we did not collect social-demographic information of the enrolled subjects, we were unable to determine epidemiological links between the clustered individuals. Other limitations were that the sample collection was confined to new infections diagnosed prospectively over a short time period with the majority of samples coming from one remote district. This might thus have biased the results towards detecting more recent transmissions in that district. We however believe most of the clustered isolates do indicate the presence of recent TB transmission. A recent study by Teo et al., examining the determinants of delayed diagnosis and treatment in Cambodia identified individuals living in rural areas were found to be significantly associated with longer time to diagnosis of tuberculosis^22^. This might thus explain the higher rates of transmission identified in Takeo, a predominantly rural region as compared to Phenom Penh, the capital of Cambodia.

In our study, while we did not identify any phenotypic resistance to rifampicin (RIF) and ethambutol (EMB), there were a handful of isolates that were resistant to isoniazid (INH) and/or streptomycin (STM) which could be expected since both INH and STM had been in clinical use for TB treatment since the 1950s^23,24^. To understand the role of specific mutations in conferring resistance to TB drugs, more data correlating WGS and phenotypic testing are required, particularly from low and middle income countries from Asia and Africa to expand the current WHO TB mutation catalogue^11^.

## Conclusion

This study shows the relevance and utility of WGS in predicting drug resistance and inference of disease transmission in the unique setting of Cambodia. Detailed genomic information about MTB strains circulating in the area with a high TB yet low MDR-TB burden might add to a better understanding of genetic markers and mechanisms producing the drug resistance in MTB elsewhere.

## Methods

### Participants and sputum sampling

TB patients were identified by Operation ASHA (OpASHA), an international NGO with a representation in Cambodia, during routine community screening procedure in the operational districts of Daun Keo (Takeo province), Dangkor and Pou Senchey (Phnom Penh capital). In brief, OpASHA staff members would visit several households a week in a village at random or guided by other community members/confirmed TB patients in the same area. At the screened households, an OpASHA member would ask if anyone in the household had typical TB symptoms. If yes, the OpASHA staff would collect a spot sputum sample and a second morning sputum sample. Both samples were delivered to the nearest authorized TB laboratory. The results were then communicated back to OpASHA. If the sputum was smear positive, the patient would be registered as a new, bacteriologically confirmed TB patient and received DOTS. The first dose was given at a health center, while the following doses were delivered by OpASHA staff to the patient’s home.

Consenting study participants older than 21 years of age with newly diagnosed, bacteriologically confirmed TB as described above would have a sputum specimen collected by the OpASHA staff before the initiation of TB treatment. The sputum specimens were collected in sterile screw-cap containers, wrapped with Parafilm™ around the lid, and transported on the same day to the local OpASHA office where they were stored at 4 °C up to 1 week before transported to the main OpASHA office in Phnom Penh where specimens were stored at 4 °C for up to 3 more weeks before shipped in batches to the Biosafety Laboratory Level 3 (BSL3) facility at the National University of Singapore (NUS).

### Ethics declaration

Informed consent was obtained from all study subjects and all experiments were performed in accordance with relevant guidelines and regulations. Ethical approval for this study was obtained from the National Ethics Committee for Health Research (NECHR) (NECHR reference: 154NECHR), Cambodia and National University of Singapore Institutional Review Board (NUS IRB reference: B-15-096E).

### Drug susceptibility testing with the BACTEC™ MGIT™ 960 system

Isolation of *Mycobacterium* strains and susceptibility tests for the TB drugs were carried out according to the MGIT manual by FIND (Geneva, Switzerland). Briefly, sputum samples (kept at 4 °C for 1–2 months) were processed with NaOH-NALC procedure (at final concentration of NaOH 1.25–2.16%), inoculated onto Middlebrook 7H11 Agar (Beckton Dickinson, Difco) or into BACTEC MGIT tube (with PANTA) with the BACTEC MGIT 960 System (Becton Dickinson, Sparks, Md., USA) and cultured at 37 °C for up to 42 days. Bacterial isolates were passage into a fresh MGIT tube (without PANTA) and drug susceptibility test was carried out according to the manufacturer’s instruction of BACTEC SIRE kit, including isoniazid (INH, 0.1 μg/ml), rifampin (RIF, 1.0 μg/ml), ethambutol (EMB, 5.0 μg/ml) and streptomycin (STR, 1.0 μg/ml).

### Genomic DNA extraction and whole-genome sequencing

Mycobacterium isolates were cultured in Middlebrook 7H9 (Beckton Dickinson, Difco) complete broth medium supplemented with 0.05% Tween 80, 0.2% glycerol, and 10% albumin-dextrose-catalase (ADC, Beckton Dickinson, Difco) at 37 °C. Bacterial cultures were centrifuged at 3000*xg* for 10 min and re-suspended into 1 mL lysis buffer (100 mM Tris–HCl (pH8.0), 250 mM NaCl, 1 mM CaCl_2_ and 3% SDS) and heat-inactivated at 95 °C for 30 min. Heat-inactivated samples were transferred to BSL2 lab, added 20 µl of Proteinase K (10 mg/mL) and incubated for 30 min at 56 °C. Samples were placed into the 2 mL tube with lysing matrix B (MP biomedicals) and bead-beat (2 cycles of 6 m/sec, 45 s) with FastPrep-24 5G (MP biologicals). Bacterial cell lysates were transferred into a new microcentrifuge tubes and genomic DNA was purified with standard phenol–chloroform DNA extraction procedure. Purified DNA was analyzed with NanoDrop 2000 spectrophotometer (Thermo Scientific) and agarose gel electrophoresis.

The genomic DNA of the samples were first fragmented using Covaris prior to library preparation using a commercially available kit, NEBNext® Ultra™ DNA Library Prep Kit for Illumina® following the manufacturer’s protocol. The samples were then pooled to be sequenced in 1 lane of a standard Illumina HiSeq4000 2 × 151 bp (multiplexed) run at the Genome Institute of Singapore.

### Bioinformatics processing for genotypic prediction of drug-susceptibilities, TB lineages, population structure and transmission cluster analysis

The generated raw sequencing data in FASTQ format were filtered using Trimmomatic^25^ to remove adapters and poor-quality bases, where the trimmed reads were subsequently mapped to the *Mycobacterium tuberculosis* H37Rv reference genome (NC_000962.3) using BWA^26^. The Genome Analysis Toolkit (GATK)^27^ was then used to correct read mapping artifacts around gaps, followed by genotype calling using SAMTools mpileup and BCFTools^28^. Homozygous single nucleotide polymorphisms (SNPs) were identified as having a minimum read depth of ~ 7X and supported by at least 75% of the reads mapped to that loci. Species identification was performed using Kraken^29^ for samples that had low mapping coverage to the H37Rv reference genome. Genotypic prediction of TB drug resistance was based on a catalogue of genetic mutations in genes reported to be associated with resistance to streptomycin (gid, *rpsL, rrs*), isoniazid (*ahpC*, *inhA*, *fabG1*, *katG*, *ndh*), rifampicin (*rpoB*) and ethambutol (*embA*, *embB*, *embC*).

The TB lineages for each isolate was predicted using 4 approaches: (1) the 62-SNP barcode as proposed by Coll et al.^12^; (2) sub-lineages 1 barcoding as proposed by Netikul et al.^13^; (3) spoligotypes using SpoTyping^30^ and (4) regions of deletions identified using RD-Analzyer^31^. Given that multiple SNPs have been identified as lineage barcodes for each sub-lineage within Netikul et al., we identified an isolate to be of the particular sub-lineage if one or more lineage-barcode SNPs are found, and the isolate did not harbour any lineage-barcode SNPs for other sub-lineages. An in-house Perl script was used to extract the identified SNPs from the isolates, excluding those in the PE/PPE genes and repeats regions to generate a multiple sequence alignment which was used as input for phylogenetic tree construction using RAxML^32^ with 1000 bootstraps under the GTRCAT model of evolution. The final maximum likelihood tree was verified using the GAMMA model of evolution by RAxML, where the phylogeny was midpoint rooted and visualized with the R package ggtree^33^. Clustering of TB isolates was inferred by pairwise comparison of the consensus genomes obtained for each TB isolate, where any pair of TB isolates having a SNP difference  ≤ 10 would be grouped in a cluster that could potentially indicate recent transmission.

## Supplementary Information

Supplementary Tables.

## Data Availability

The *Mycobacterium tuberculosis* whole-genome sequencing data will be deposited in the public archive of short-read archive (SRA) under the BioProject ID PRJNA756844.
